# Adaptation, psychometric properties and factor structure of the Spanish Quality in Psychiatric Care-Outpatient Staff (QPC-OPS) instrument

**DOI:** 10.1038/s41598-022-08039-w

**Published:** 2022-03-07

**Authors:** Manuel Tomás-Jiménez, Juan Roldán-Merino, Sara Sanchez-Balcells, Agneta Schröder, Lars-Olov Lundqvist, Montserrat Puig-Llobet, Antonio R. Moreno-Poyato, Marta Domínguez del Campo, Maria Teresa Lluch-Canut

**Affiliations:** 1 Parc Sanitari Sant Joan de Déu, Sant Boi de Llobregat, Barcelona Spain; 2 Mental Health Department, Campus Docent Sant Joan de Déu-Private Foundation, University of Barcelona, Edifici Esade-3, C/Miret I Sans, 10-16, 08034 Barcelona, Spain; 3 University Health Care Research Center, Faculty of Medicine and Health, Örebro University, Örebro, Sweden; 4 Department of Nursing, Faculty of Health Care and Nursing, Norwegian University of Science and Technology (NTNU), Trondheim, Norway; 5 Public Health, Mental Health and Maternal-Infant Nursing Department, Nursing College, University of Barcelona, Health Sciences Campus Bellvitge, Hospitalet de Llobregat, Barcelona, Spain

**Keywords:** Psychology, Health care

## Abstract

Quality of care is a multidimensional concept that should include the perspectives of all parties involved. There are few instruments with adequate psychometric properties for the assessment of the quality of psychiatric care in community mental health. *Quality in Psychiatric Care-Outpatient Staff (QPC-OPS)* instrument has adequate psychometric properties to evaluate the quality of psychiatric care from the perspective of professionals. The aim of this study was to validate the Spanish version of the *QPC-OPS* instrument. The instrument was translated and back-translated, and then was administered to 260 professionals from distinct community mental health services. To assess test–retest reliability, it was re-administered after 7–14 days (n = 157). Confirmatory factor analysis revealed an 8-factor-structure identical to the original version, showing the good fit of the model. The internal consistency coefficient (Cronbach’s alpha) was 0.885. The intraclass correlation coefficient was 0.847 (95% IC 0.790–0.888), which was higher than 0.70 in all factors bar one. The NT394 General Satisfaction Scale was used for analysis of convergent validity showing a rho correlation of 0.31 (p < 0.0001). Results show that the Spanish version of the QPC-OPS instrument is valid and reliable for the assessment of the quality of psychiatric care in the community setting.

## Introduction

Since the year 1986, with General Health Law 14/1986 of the 25th April, the Spanish Ministry of Health has focused its efforts on promoting community care in mental health and today this remains one of the pillars of mental health strategies in the National Health System quality plan. This aims to avoid segregating those receiving care from the community and to provide care in a location close to their home^1,2^.

Quality of care can be defined in several ways. Donabedian maintains that the essence of care lies in the balance between benefit and harm^3^. Later authors, however, defined quality of care in terms of care efficiency and access to health resources for both users and their families^4,5^. The concept of quality of care, within current standards in the field of mental health, includes the therapeutic setting, the assessment, the therapeutic relationship and counseling, professional performance, practice evaluation, and environmental health as key criteria in quality^6,7^.

Quality of care is a multidimensional concept^8^ that is perceived by users as something positive^9^. However, unlike the concept of patient satisfaction, quality of care must include the perspectives of all parties involved^10^. However, job satisfaction is an element to take into account in the assessment of professionals, given the influence it can have not only on their perception of their health but also on the performance of their work^11^. Of the instruments used in the assessment of job satisfaction, the NTP 394 scale created by Warr et al.^11^ and subsequently validated in Spanish by Pérez and Fidalgo^12^, stands out as it is one of the most frequently used instruments in our setting for the evaluation of job satisfaction in various environments, including health care^13^.

Professionals have already been described as an essential element in ensuring high standards of quality of care in current approaches such as person-centered care^14^. The combination of the perspectives of professionals and users in the community setting is necessary and provides complementary information of great value^15^. Consequently, the perspective of mental health professionals should be taken into account and used as an additional indicator of care quality in mental health^10,16^. This would help to identify quality improvement strategies^17^ and to detect factors that can have a negative impact on the quality of care, such as burnout or work overload among care professionals^18–20^. Nevertheless, it has been observed that professionals from distinct disciplines involved in psychiatric care have differing points of view on what characterizes quality of care^4,21,22^.

Separately, there is a lack of comparative studies on the perceptions of quality of care among patients and professionals^23^, mainly due to the absence of standardized assessment instruments with adequate psychometric properties^18^. By way of illustration, a recent systematic review revealed that despite the considerable volume of existing instruments that evaluate the quality of psychiatric care or satisfaction with psychiatric care, their psychometric properties were not very robust and some validation processes involving these instruments were not completely satisfactory^24^.

One instrument that does possess adequate psychometric properties for the community mental health context is the Psychiatric Out-Patient Experiences Questionnaire (POPEQ). However, it only examines the user’s perspective^25^.

The only instrument found to have adequate psychometric properties applicable in the community mental health context, and which assesses the quality of psychiatric care from the perspective of both professionals and users, is the Quality in Psychiatric Care^26^ in its two versions: Quality in Psychiatric Care-Outpatient (QPC-OP) and Quality in Psychiatric Care-Outpatient Staff (QPC-OPS). The latter version has recently been validated in Norwegian^27^.

This instrument forms part of the family of instruments Quality in Psychiatric Care (QPC) that assess the quality of psychiatric care through its multiple versions in the hospital setting (Quality in Psychiatric Care-In-patients—QPC-IP)^28^, the community (Quality in Psychiatric Care-Outpatient—QPC-OP)^26^ and forensic (Quality in Psychiatric Care-Forensic In-patient QPC-FIP)^29^. The definition of care used to create the instrument was developed through a phenomenographic study^9^, it was evaluated for face validity in a pilot study and empirically tested in a sample of patients admitted to psychiatric hospital units in Sweden^30^.

This study is part of a wider international project to adapt the QPC-OPS instrument in a number of countries, test the psychometric properties and dimensional equivalence of the different versions according to language, and describe and compare the quality of psychiatric care in the community setting in these countries. In this context, the aim of the study was to adapt the QPC-OPS instrument into Spanish and analyze its reliability and validity.

## Methods

### Design

The study was conducted in two phases. In the first phase, the translation and adaptation of the QPC-OPS instrument into Spanish was carried out. In the second phase, the psychometric properties of the Spanish version of the QPC-OPS instrument were analyzed.

### Participants and study setting (sample size)

The sample consisted of 260 professionals from different disciplines who work in community mental health services (nursing, psychiatry, social education, case management, social work, administration and occupational therapy), who were actively working in a community service at the time of the study (Outpatient Mental Health Center, Day Hospital, Labor Reintegration Service, Community Rehabilitation Service) and who participate voluntarily. Having less than one year’s experience in the area of mental health was established as an exclusion criterion. Non-probability convenience sampling was used.

Data collection was carried out between February, 2019 and February, 2020.

Calculation of the sample size was based on internal consistency, temporal stability and construct validity. Estimation of internal consistency was performed following the recommendations of Streiner et al., who considered that between 5 and 20 individuals should be included for each instrument item^31^. In this study, we agreed to include a minimum of five individuals for each item.

To analyze temporal stability, it was estimated that a minimum of 61 professionals would be needed to detect an intraclass correlation coefficient (ICC) around 0.70 between two administrations of the instrument, assuming a confidence level of 95% and a power of 80% in a bilateral comparison^32^.

For construct validity, it was established that the minimum number of subjects necessary would be 250^33^.

### Variables and sources of information

As indicated, the QPC-OPS instrument assesses the quality of psychiatric care in the community setting from the perspective of the professionals working there.

It consists of a total of 30 items distributed across 8 factors as follows: encounter (6 items), participation-empowerment (3 items), participation-information (5 items), discharge (3 items), support (4 items), environment (3 items), next of kin (2 items), and accessibility (4 items). Each item begins with the words “I feel that…” and is scored on a Likert-type scale with four response options ranging from 1 (totally disagree) to 4 (totally agree) with a “not applicable” option for each if considered necessary. A global and per-factor score can be obtained; the maximum global score is 120 points and the minimum 30 points so that a high score on each factor or globally indicates a good perception of the quality of psychiatric care on the part of the professionals. Conversely, a low score justifies the need for an intervention designed to bring about improvements in the areas identified.

The General Satisfaction scale NTP 394 created by Warr et al.^11^ and validated in Spanish by Pérez and Fidalgo^12^ was used to analyze convergent validity. The Spanish version showed a Cronbach’s alpha (α) coefficient that ranged between 0.85 and 0.88 and an Intraclass Correlation Coefficient (ICC) of 0.63. This scale assesses intrinsic and extrinsic aspects of working conditions and consists of 15 items. Each item is evaluated through an ordinal scale that ranges from 1 (very dissatisfied) to 7 (very satisfied). The total score is obtained by summing the items, producing a final score between 15 and 105.

In addition, data were collected on other variables related to sociodemographic and labor characteristics of the sample: age, sex, nationality, professional category, service where currently working and number of years in the service.

### Procedure

The translation and back-translation process was carried out following the Standards for Educational and Psychological Testing^34^.

First, the original version was translated into Spanish by two independent native-speaker translators who had no knowledge of the instrument or the aims of the study. A group of experts comprising nurses, psychiatrists and psychologists reviewed both translations and reached agreement on the first version of the instrument in Spanish. Subsequently, the Spanish version was back-translated into the original language to confirm that the Spanish translation corresponded to the original version in Swedish. In addition, the original authors of the QPC-OPS examined the back-translation and compared it with the original version, finding no discrepancies requiring modification. This version was then pilot tested in 30 professionals with the aim of assessing item comprehension and clarity, as well as the time needed to administer it. Following the debriefing, it was not considered necessary to make any changes to either format of content.

### Statistical analysis

#### Construct validity

Construct validity was analyzed through confirmatory factor analysis (CFA) with estimated parameters using the method of least squares generalized with a polychoric correlation matrix. This method has the same properties as the maximum likelihood estimation, even though the criteria were less strict than the normal ones. It is mainly used to measure ordinal items^35^.

The following fit indices were calculated to determine the overall fit of the model: Bentler Bonnet Normed Fit Index (BBNFI), Bentler Bonnet Non-Normed Fit Index (BBNNFI), the Goodness-of-Fit Index (GFI), the Adjusted Goodness-of-Fit Index (AGFI), the Comparative Fit Index (CFI), the Root Mean Square Error of Approximation (RMSEA), the chi-squared goodness-of-fit test and the ratio between chi-squared and the degrees of freedom (χ^2^/df). The criteria for a good fit were an X^2^/df ratio < to 3 and BBNFI, BBNNFI, GFI, AGFI and CFI close to 0.90^36–38^, and a RMSEA value lower than 0.08^39,40^.

#### Convergent validity

Convergent validity was analyzed using the Spearman correlation coefficient with the NTP 394 General Satisfaction Scale.

As a complementary method, an analysis of the Spearman correlation was conducted between the QPC-OPS questionnaire factors to assess convergent validity based on the hypothesis that the correlation between each factor and the general instrument should be higher than the correlations between the factors^41^.

#### Reliability

The item analyses included calculation of item means, standard deviations and corrected item-total correlation.

Cronbach’s alpha was used to assess the internal consistency of the instrument globally and for each of the factors. Levels of reliability above 0.70 were considered adequate^31^.

Temporal stability or test–retest was evaluated after 7–14 days through the ICC in a sample of 157 professionals. The values of this coefficient range between 0 and 1. A value greater than or equal to 0.70 was considered an indicator of good agreement^31^. Composite reliability was also calculated.

The SPSS Statistics program version 26 was used for analyses, along with EQS program version 6.2 for the confirmatory factor analysis (CFA)^42^.

### Ethics declarations

First, in order to be able to carry out the study, permission to translate and adapt the QPC-OPS instrument into Spanish was sought from the original authors.

This study was approved by the research ethics committee at Fundación Sant Joan de Déu, under code CEIC PIC-83-16. All research was performed in accordance with relevant guidelines and regulations. All participants and their guardians were informed about the aim of the study and gave verbal and written consent to voluntarily and anonymously take part.

## Results

### Participant characteristics

A total of 260 professionals participated, of whom 26.2% were men and 73.8% were women. Some 95.8% of participants had Spanish nationality and the remaining 4.2% were of different nationalities. The sample consisted of a variety of professional categories: nursing (29.61%), psychiatry (20.38%), psychology (16.53%), social education (8.84%), case management (7.7%), social work (7.30%), administration (5%) and occupational therapy (4.61%). The mean age was 40 ± 10.3 years, while the mean of years worked in community service was 8.68 ± 7.70 years.

### Construct validity

#### Confirmatory factor analysis (CFA)

Confirmatory factor analysis was used to verify the internal structure of the instrument, in which an 8-factor model identical to the structure of the original instrument was proposed. Table 1 shows the fit of the model. All indices showed a reasonable fit.

**Table 1:** Goodness-of-fit indices for the confirmatory model Spanish QPC-OPS.

Index	Value
BBNFI	0.782
BBNNFI	0.861
GFI	0.961
AGFI	0.956
CFI	0.880
RMSEA	0.060
Cronbach’s alpha	0.885
Goodness of fit test	χ^2^ = 726.045; df = 377; p < 0.0001
Adjustment reason	χ^2^/df = 1.92

*BBNFI* Bentler Bonnet Normed Fit Index, *BBNNFI* Bentler Bonnet Non-Normed Fit Index, *GFI* Goodness of Fit Index, *AGFI* Adjusted Goodness of Fit Index, *CFI* Comparative Fit Index, *RMSEA* Root Mean Square Error of Approximation, *df* Degrees of freedom.

**Figure 1:**
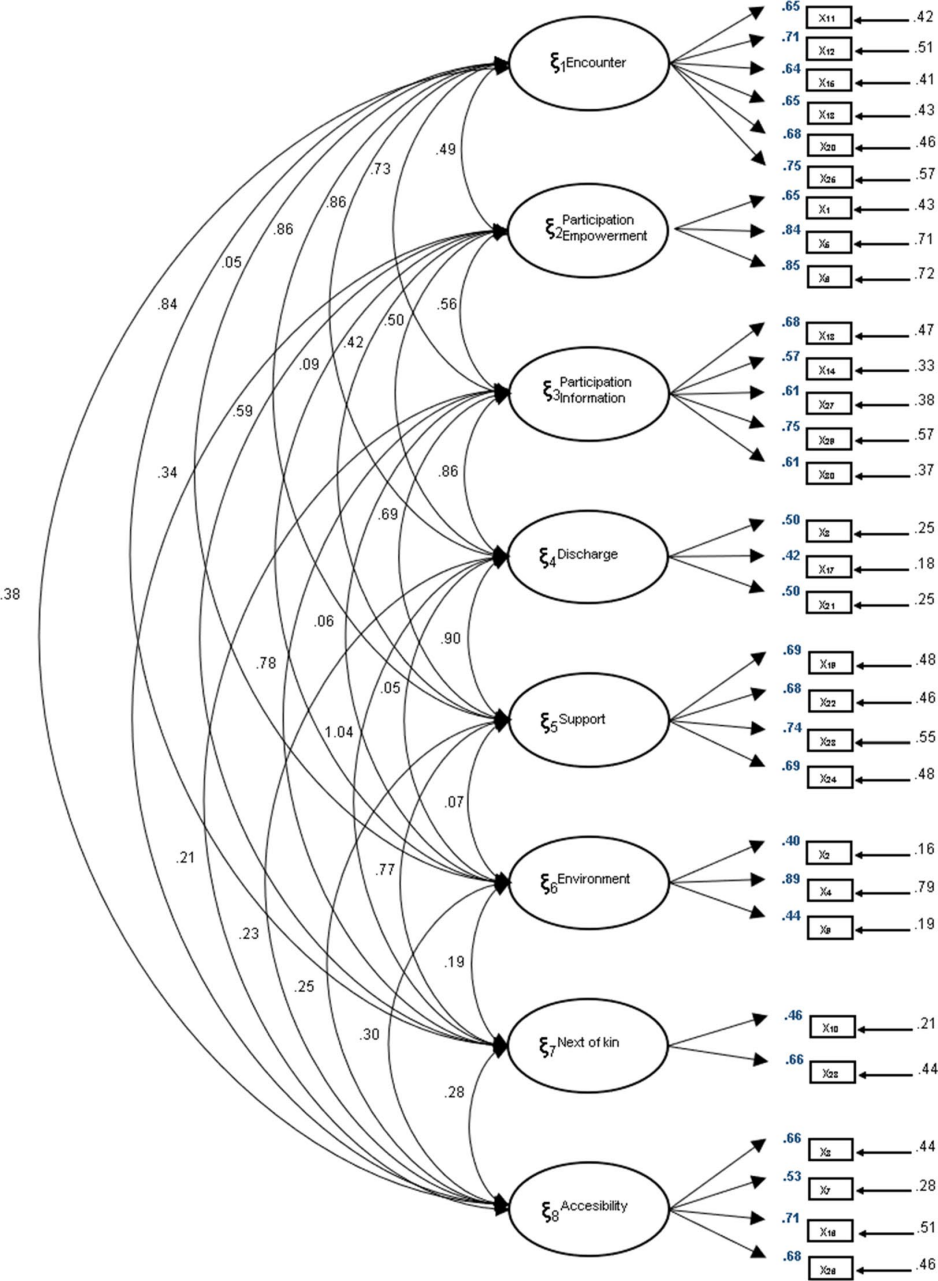
Factor loadings derived from the least square estimation (least squares). Confirmatory factor analysis (λ_ij_).

All item saturations were equal to or greater than 0.50 with the exception of items 2 (0.40), 9 (0.44), 10 (0.46) and 17 (0.42). Correlations between factors in the Spanish version of the QPC-OPS are shown in Fig. 1.

### Convergent validity

Convergent validity was analyzed using the Spearman correlation coefficient with the NTP 394 General Satisfaction Scale. The correlation obtained was rho = 0.31 (p < 0.0001).

The hypothesis was confirmed in the analysis of the correlations between the factors and the general instrument, with the strongest correlations found between the majority of factors and the general instrument. Factors 1 (*Encounter*) and 3 (*Participation-Information*) showed the strongest correlations with the total instrument (rho = 0.764 y rho = 0.755, respectively), while Factor 6 (*Environment*) had the weakest correlation (rho = 0.348). Table 2 shows the correlations of all the factors with the total instrument score.

**Table 2:** Correlations between Spanish QPC-OPS factors and total instrument.

	F1	F2	F3	F4	F5	F6	F7	F8	Total
F1. Encounter	1.000								
F2. Participation—Empowerment	0.365*	1.000							
F3. Participation—Information	0.603*	0.401*	1.000						
F4. Discharge	0.491*	0.311*	0.508*	1.000					
F5. Support	0.636*	0.309*	0.555*	0.506*	1.000				
F6. Environment	0.102	0.105	0.055	0.069	0.073	1.000			
F7. Next of kin	0.451*	0.354*	0.447*	0.439*	0.384*	0.151*	1.000		
F8. Accessibility	0.316*	0.320*	0.213*	0.132*	0.218*	0.197*	0.193*	1.000	
Total Spanish QPC-OPS	0.764*	0.616*	0.755*	0.612*	0.681*	0.348*	0.589*	0.593*	1.000

n = 260.

*All correlation coefficients are significant at p < 0.001.

### Reliability

Cronbach’s alpha internal consistency coefficient for the whole instrument was 0.885, reaching values above 0.70 in five of the eight factors (Table 3). For the factors *F4.Discharge, F6.Environment* and *F7.Next of Kin*, Cronbach’s alpha values of 0.462, 0.537 and 0.440 were obtained, respectively. Internal consistency was not notably improved by excluding any of the items.

**Table 3:** Spanish QPC-OPS. Test–retest ICC (n = 157), Composite reliability and Cronbach’s alpha.

Factors	ICC (CI 95%)	Composite reliability	Cronbach’s alpha
F1. Encounter	0.771 (0.686–0.833)	0.842	0.843
F2. Participation—Empowerment	0.805 (0.732–0.857)	0.831	0.827
F3. Participation—Information	0.843 (0.784–0.885)	0.787	0.784
F4. Discharge	0.708 (0.601–0.787)	0.473	0.462
F5. Support	0.681 (0.562–0.767)	0.800	0.799
F6. Environment	0.840 (0.780–0.883)	0.621	0.537
F7. Next of kin	0.779 (0.697–0.838)	0.488	0.440
F8. Accessibility	0.873 (0.826–0.907)	0.748	0.734
Total	0.847 (0.790–0.888)	0.957	0.885

*ICC* Intraclass Correlation Coefficient, *CI* Confidence Interval.

The mean item value ranged from 2.58 to 3.82 and the standard deviation ranged from 0.44 to 0.92. Only three items showed an item–total correlation under 0.20 (item 2: *“Security in the center is high”*, item 4: *“Patients can feel safe along with other patients in the waiting room”* and item 9: *“Patients are not bothered by other patients in the waiting room”*) (Table 4).

**Table 4:** Descriptive Statistics of Scale QPC-OPS Items. Item mean, standard deviations and corrected item-total correlation.

Summary of the contents of the items	Mean	SD	Corrected item-total correlation	Cronbach’s alpha Total instrument without item
Item 1. Patients decide on their care and treatment	2.89	0.63	0.408	0.882
Item 2. Security in the center is high	2.58	0.92	0.100	0.893
Item 3. Patients can easily access professionals by phone	3.28	0.81	0.293	0.886
Item 4. Patients can feel safe along with other patients in the waiting room	3.30	0.62	0.178	0.887
Item 5. The opinion of patients is respected when establishing their care and treatment	3.27	0.64	0.533	0.879
Item 6. Patients participate in decisions about their care	3.17	0.64	0.526	0.880
Item 7. It is easy for patients to get an appointment with professionals	3.27	0.79	0.394	0.883
Item 8. The care and treatment patients receive is effective and helps them with their problems	3.30	0.52	0.414	0.882
Item 9. Patients are not bothered by other patients in the waiting room	3.10	0.64	0.127	0.888
Item 10. Family members or friends of patients are offered the opportunity to participate in their care and treatment to the extent they wish	3.27	0.66	0.401	0.882
Item 11. Staff treat patients with consideration and proximity	3.77	0.44	0.549	0.880
Item 12. Professionals worry about knowing why the patient is angry or irritated	3.73	0.47	0.609	0.879
Item 13. The patient's experience is taken into account in order to establish medical treatment	3.42	0.66	0.555	0.879
Item 14. Patients are offered health education to learn how to identify symptoms of worsening disease	3.56	0.60	0.447	0.881
Item 15. Professionals treat patients with respect	3.82	0.45	0.536	0.880
Item 16. It's easy to contact the center by phone	3.17	0.88	0.323	0.886
Item 17. Patients are helped to look for work or other occupations	3.45	0.64	0.363	0.883
Item 18. Professionals understand patients' feelings	3.50	0.56	0.548	0.879
Item 19. Professionals offer strategies to patients to prevent them from harming the people around them, if they had those ideas at all	3.60	0.52	0.555	0.880
Item 20. Professionals spend time listening to patients	3.57	0.58	0.593	0.878
Item 21. Patients receive information on where they can go if they need help after contact with the center is over	3.65	0.51	0.436	0.882
Item 22. Professionals offer strategies to patients to prevent them from harming themselves if they had such ideas	3.63	0.52	0.529	0.880
Item 23. Professionals help patients understand that it is not shameful to have a mental illness	3.67	0.52	0.588	0.879
Item 24. Professionals help the patient understand that feelings of guilt and shame should not prevent them from asking for help	3.67	0.52	0.534	0.880
Item 25. Professionals care about the care and treatment of patients	3.72	0.49	0.643	0.878
Item 26. It is easy to establish telephone contact with the doctor responsible for the patient's care and treatment	2.95	0.78	0.336	0.884
Item 27. The patient is clearly informed about their mental illness and diagnosis	3.31	0.63	0.472	0.881
Item 28. Patients' family members are treated with respect	3.80	0.42	0.557	0.880
Item 29. Patients are offered information about their disease so they can participate in their treatment	3.47	0.61	0.577	0.879
Item 30. Patients are given information about different treatment options so they can decide which one they think is best suited	3.03	0.71	0.472	0.881
Overall instrument			101.91	9.08

*SD* Standard Deviation.

Appendix 1 shows inter-correlations between individual items for each QPC-OPS sub-scale. In factor 6 (*Environment*) the correlation between items 2 and 9 was 0.130.

The ICC analysis demonstrated that test–retest reliability was 0.847 (95% CI 0.790–0.888; n = 157), and this value was higher than 0.70 in all instrument factors, except D5 with a value of 0.681 (Table 3). Composite reliability was 0.957 for the whole instrument, reaching values higher than 0.70 in five of the eight factors. For the factors *F4.Discharge, F6.Environment* and *F7.Next of Kin*, composite reliability values of 0.473, 0.621 and 0.488 were obtained, respectively (Table 3).

## Discussion

The aim of this study was to adapt the Quality in Psychiatric Care Outpatient Staff (QPC-OPS) instrument to Spanish and analyze its reliability and validity. This instrument allows assessment of the quality of community psychiatric care from the professionals’ perspective so that strong points and areas for improvements in care can be detected. On the other hand, it is a useful instrument for the evaluation of interventions focused on improving the quality of psychiatric care.

The adaptation process of the QPC-OPS involved a translation and back-translation to obtain the Spanish version. Other studies in the QPC family^27,43–48^ produced versions in other languages using a similar process. Results in this phase were satisfactory and there were no difficulties found with respect to the comprehension and administration of the instrument.

Results show that, at the psychometric level, the values for construct validity (Confirmatory factor analysis), internal consistency and temporal stability (test–retest) were adequate.

The confirmatory analysis performed that the Spanish version, like the Norwegian version^27^, has the same 8 factors on quality of care as the original Swedish QPC-OP^26^ version and no modification of any item was required.

For the analysis of reliability, internal consistency was analyzed using Cronbach’s alpha. A Cronbach’s alpha of 0.885 was obtained for the whole instrument and the factors in general showed values greater than 0.70, which is considered an adequate value according to Nunnally and Berstein^49^. The global Cronbach’s alpha is somewhat lower than the original version of the instrument^26^ and other versions of the QPC family^28,29,43,44,48^, although higher than the Norwegian community version^27^ and the hospital version for Indonesian users^47^ with a value identical to the hospital version for Indonesian professionals^46^. Cronbach’s alpha values lower than 0.70 were found for the factors *F4.Discharge* (0.462), *F6.Environment* (0.537) and *F7.Next of kin* (0.440), which are considered less adequate values. These low values are, in all likelihood, due to the reduced number of items that make up these factors (*F4. Discharge*: 3 items, *F6.Environment*: 3 items and *F7.Next of kin*: 2 items). Some of the inter-correlations between the individual items were low. Future research should assess the possibility of identifying other items that may better represent these factors to improve the psychometric properties.

In the original Swedish QPC-OP version^26^, the factors *F4.Discharge* and *F7.Next of kin* obtained values below 0.70, and the *F6.Environment* factor showed a value very close to 0.70.

Temporal or test–retest stability was also analysed in the study. This value was not calculated in the original Swedish QPC-OP version^26^, the other original versions of the QPC family^28,29^ or in the translated versions^44,46,47^, with the exception of the Spanish hospital versions of the QPC-IPS and QPC-IP^43,48^. The ICC for the whole instrument and for each of the factors was above 0.70 except in the case of *F5.Support* (0.681), which in general is considered good agreement^31,32^. For the Spanish QPC-IPS and QPC-IP, the ICC for the whole instrument was 0.91 and the factors had values above 0.70 except for the factor *F6.Discharge*^43,48^.

Convergent validity of the Spanish QPC-OPS in our study was calculated through the Spearman rho correlation coefficient with the NTP 394 General Satisfaction Scale. The correlation obtained was positive^50^, showing that the greater the job satisfaction, the higher the perception of the quality of psychiatric care provided. Of the studies related to the family of QPC instruments, this is the third study in which convergent validity is calculated, the first being the Spanish hospital version of the QPC-IPS^43^ and the second the Spanish hospital version of the QPC-IP^48^. The Spanish QPC-IPS calculated convergent validity using the same NTP 394 scale, obtaining a higher value (rho = 0.58); considered a moderate positive correlation. The Spanish QPC-IP calculated convergent validity using a 10-point numerical satisfaction scale, obtaining also a higher value (rho: 0.49); considered a moderate positive correlation.

At the same time, the correlation was analyzed between the total instrument score and each of its factors, showing that the correlation was higher between each factor and the general instrument than the correlations between the factors. This confirms the Fayer and Machin hypothesis^41^.

The limitations of this study arise from the lack of uniformity in the professional categories as each one has differing numbers of professionals and the concept of “community mental health professionals” is very wide, encompassing many health workers. However, this reflects the reality of the composition of Spanish community care teams. A further limitation is that this instrument was adapted in the community context so it should only be used in this population profile or a similar one. Another limitation that should be highlighted is that the NTP scale presented a low correlation with the total QPC-OPS score. This may be due to the fact that it is not the most appropriate gold standard for the assessment of the quality of care. Nevertheless, it is a widely-used instrument in our setting. Future research should take into account that there is a need for a more suitable instrument to function as the gold standard.

Finally, it should be emphasized that it was not possible to assess sensitivity to change or predictive validity given the cross-sectional design of the study. These limitations should be taken into account in the design of future studies.

## Conclusions

The Spanish version of the QPC-OPS instrument is a simple, useful tool for the measurement of various aspects of the quality of community psychiatric care from the perspective of mental health professionals. Its 8-factor structure and psychometric properties are consistent and in agreement with the original version, allowing the instrument to be used to measure the quality of community psychiatric care from the perspective of professionals in the Spanish-speaking population. The results of these measurements can be used to increase the capacity to assess the quality of services provided.

## Supplementary Information

Supplementary Information.

## Data Availability

The data that support the findings of this study are available upon reasonable request from the corresponding author. The data are not publicly available due to privacy and ethical restrictions. The data was taken from our own study.
