# Supplementary material for: Adaptation, psychometric properties and factor structure of the Spanish Quality in Psychiatric Care-Outpatient Staff (QPC-OPS) instrument
Source: Sci Rep. 2022 Mar 7;12:4018. doi: 10.1038/s41598-022-08039-w (PMC8901654; doi:10.1038/s41598-022-08039-w)
Supplement: Supplementary file 1 — Supplementary Information. [file 41598_2022_8039_MOESM1_ESM.docx]

***Adaptation, psychometric properties and factor structure of the Spanish Quality in Psychiatric Care – Outpatient Staff (QPC-OPS) instrument .***

Manuel Tomás-Jiménez, Juan Roldán-Merino*, Sara Sanchez-Balcells, Agneta Schröder, Lars-Olov Lundqvist, Montserrat Puig-Llobet, Antonio R Moreno-Poyato, Marta Domínguez del Campo, Maria Teresa Lluch-Canut

*Appendix 1.*

*Inter-element (items) correlation table for Factor 1. Encounter*

| Inter-element correlations matrix | | | | | | | |
| --- | --- | --- | --- | --- | --- | --- | --- |
|  | **Item 11** | **Item 12** | **Item 15** | **Item 18** | **Item 20** | **Item 25** |  |
| **Item 11.** Staff treat patients with consideration and proximity | 1.000 |  |  |  |  |  |  |
| **Item 12.** Professionals worry about knowing why the patient is angry or irritated | .638 | 1.000 |  |  |  |  |  |
| **Item 15**. Professionals treat patients with respect | .669 | .596 | 1.000 |  |  |  |  |
| **Item 18**. Professionals understand patients' feelings | .424 | .437 | .437 | 1.000 |  |  |  |
| **Item 20.** Professionals spend time listening to patients | .437 | .427 | .422 | .330 | 1.000 |  |  |
| **Item 25.** Professionals care about the care and treatment of patients | .493 | .455 | .509 | .507 | .536 | 1.000 |  |

*Inter-elements (items) correlation table for Factor 2. Participation-Empowerment*

| Inter-element correlations matrix | | | |
| --- | --- | --- | --- |
|  | **Item 1** | **Item 5** | **Item 6** |
| **Item 1.** Patients decide on their care and treatment | 1.000 |  |  |
| **Item 5.** The opinion of patients is respected when establishing their care and treatment | .552 | 1.000 |  |
| **Item 6.** Patients participate in decisions about their care | .580 | .709 | 1.000 |

*Inter-element (items) correlation table for Factor 3. Participation-Information*

| Inter-element correlations matrix | | | | | |
| --- | --- | --- | --- | --- | --- |
|  | **Item 13** | **Item 14** | **Item 27** | **Item 29** | **Item 30** |
| **Item 13.** The patient's experience is taken into account in order to establish medical treatment | 1.000 |  |  |  |  |
| **Item 14.** Patients are offered health education to learn how to identify symptoms of worsening disease | .385 | 1.000 |  |  |  |
| **Item 27.** The patient is clearly informed about their mental illness and diagnosis | .338 | .395 | 1.000 |  |  |
| **Item 29.** Patients are offered information about their disease so they can participate in their treatment | .424 | .472 | .646 | 1.000 |  |
| **Item 30.** Patients are given information about different treatment options so they can decide which one they think is best suited | .363 | .267 | .418 | .535 | 1.000 |

*Inter-element (items) correlation table for Factor 4. Discharge*

| Inter-elements correlations matrix | | | |
| --- | --- | --- | --- |
|  | **Item 8** | **Item 17** | **Item 21** |
| **Item 8.** The care and treatment patients receive is effective and helps them with their problems | 1.000 |  |  |
| **Item 17.** Patients are helped to look for work or other occupations | .231 | 1.000 |  |
| **Item 21.** Patients receive information on where they can go if they need help after contact with the center is over | .226 | .223 | 1.000 |

*Inter-element (items) correlation table for Factor 5. Support*

| Inter-element correlations matrix | | | | |
| --- | --- | --- | --- | --- |
|  | **Item 19** | **Item 22** | **Item 23** | **Item 24** |
| **Item 19.** Professionals offer strategies to patients to prevent them from harming the people around them, if they had those ideas at all | 1.000 |  |  |  |
| **Item 22.** Professionals offer strategies to patients to prevent them from harming themselves if they had such ideas | .532 | 1.000 |  |  |
| **Item 23.** Professionals help patients understand that it is not shameful to have a mental illness | .422 | .431 | 1.000 |  |
| **Item 24.** Professionals help the patient understand that feelings of guilt and shame should not prevent them from asking for help | .429 | .508 | .671 | 1.000 |

*Inter-element (items) correlation table for Factor 6. Environment*

| Inter-elements correlations matrix | | | |
| --- | --- | --- | --- |
|  | **Item 2** | **Item 4** | **Item 9** |
| **Item 2.** Security in the center is high | 1.000 |  |  |
| **Item 4.** Patients can feel safe along with other patients in the waiting room | .343 | 1.000 |  |
| **Item 9.** Patients are not bothered by other patients in the waiting room | .130 | .469 | 1.000 |

*Inter-element (items) correlation table for Factor 7. Next of kin*

| Inter-element correlations matrix | | |
| --- | --- | --- |
|  | **Item 10** | **Item 28** |
| **Item 10.** Family members or friends of patients are offered the opportunity to participate in their care and treatment to the extent they wish | 1.000 |  |
| **Item 28.** Patients' family members are treated with respect | .310 | 1.000 |

*Inter-element (items) correlation table for Factor 8. Accessibility*

| Inter-element correlations matrix | | | | |
| --- | --- | --- | --- | --- |
|  | **Item 3** | **Item 7** | **Item 16** | **Item 26** |
| **Item 3.** Patients can easily access professionals by phone | 1.000 |  |  |  |
| **Item 7.** It is easy for patients to get an appointment with professionals | .269 | 1.000 |  |  |
| **Item 16.** It's easy to contact the center by phone | .571 | .280 | 1.000 |  |
| **Item 26.** It is easy to establish telephone contact with the doctor responsible for the patient's care and treatment | .506 | .251 | .556 | 1.000 |
